# Clinical significance of NCOA5 gene rs2903908 polymorphism in Behçet's disease

**DOI:** 10.17179/excli2017-189

**Published:** 2017-05-04

**Authors:** Aydin Rustemoglu, Esra Erkol Inal, Ahmet Inanir, Duygu Ekinci, Ulker Gul, Serbulent Yigit, Omer Ates, Nevin Karakus

**Affiliations:** 1 Gaziosmanpasa University, Faculty of Medicine, Department of Medical Biology, Tokat, Turkey; 2 Suleyman Demirel University, Faculty of Medicine, Department of Physical Medicine and Rehabilitation, Isparta, Turkey; 3 Gaziosmanpasa University, Faculty of Medicine, Department of Physical Medicine and Rehabilitation, Tokat, Turkey; 4 Akdeniz University, Faculty of Medicine, Department of Dermatology, Antalya, Turkey

**Keywords:** Behçet's disease, NCOA5, rs2903908, uveitis

## Abstract

Behçet's disease (BD) is an autoimmune multisystemic disease. The precise etiology of BD is not fully understood; however, it is thought that interactions between genetic and environmental factors play an essential role in its pathogenesis. The nuclear receptor coactivator-5 (*NCOA5*) gene encodes a coregulator for nuclear receptor subfamily 1 group D member 2 (*NR1D2*) and estrogen receptor 1 and 2 (*ESR1* and *ESR2*). Also, the *NCOA5* gene insufficiency leads to an elevated expression of IL-6, and increased levels of IL-6 were found to be related to the pathogenesis of BD. In this study, we aimed to clarify the impact of the *NCOA5* rs2903908 polymorphism on susceptibility and clinical findings of BD. This study included 671 participants (300 BD patients and 371 healthy controls). The analyses of *NCOA5* rs2903908 polymorphism was performed by using the TaqMan allelic discrimination assay. The frequency of TT genotype of the *NCOA5* rs2903908 polymorphism was found significantly higher in BD patients compared to those in healthy controls (p=0.016, OR=1.46, 95 % CI=1.08-1.99). Also, the frequencies of CT genotype was observed significantly higher in BD patients with genital ulceration and uveitis than without genital ulceration and uveitis (p=0.002 and p=0.005, respectively). The most significant association was found between C allele frequencies of BD patients with and without uveitis (p=0.0001). Our study represents for the first time that the *NCOA5* rs2903908 polymorphism seemed to be linked to BD susceptibility and clinical findings.

## Introduction

Behçet's disease (BD) is a multisystemic, chronic inflammatory autoimmune disease with unknown aetiology and described as a triad of recurrent oral aphthous ulcers, genital ulceration, and uveitis. The other clinical findings include mucocutaneous, articular, neurologic, urogenital, vascular, gastrointestinal, and pulmonary involvements (Gül, 2005[12]). BD is more prevalent in the geographic region along the “Silk Road,” which lies from the Mediterranean to the Far East. The prevalence of BD in Turkey varies from 20 to 420 cases per 100,000 adults, which is the highest prevalence rate identified in the world (Idil et al., 2002[15]; Azizlerli et al., 2003[3]; Cakir et al., 2004[7]). The male-to-female ratio is nearly equal, and the disease occurs in individuals aged 18 to 40 years (Gül, 2005[12]; Alpsoy et al., 2007[1]). 

The precise aetiology of BD is not fully understood; however, it is thought that interactions between genetic and environmental factors play an essential role in the pathogenesis of the disease. Detecting interactions between the genes and mechanisms playing roles in the pathogenesis of numerous diseases can be difficult to find, but there are many examples in which common genetic factors take part in the development of several chronic inflammatory autoimmune diseases (Aune et al., 2003[2]; Lee et al., 2012[19]; Oğuz et al., 2016[23]). This suggests that certain gene regions may contribute similar pathways that are shared by various autoimmune diseases. In this field, the *CD40* gene was indicated as a susceptible gene for a number of chronic inflammatory and autoimmune diseases (García-Bermúdez et al., 2012[11]; Joo et al., 2013[17]). Additionally, *CD40* is a member of tumor necrosis factor receptor superfamily (Foy et al., 1996[9]). The nuclear receptor coactivator-5 (*NCOA5*) gene, located on the 20q13.1 region (a 400-kb surrounding zone of the *CD40* gene), encodes a coregulator for nuclear receptor subfamily 1 group D member 2 (*NR1D2*) and estrogen receptor 1 and 2 (*ESR1* and *ESR2*). *NCOA5* is known to modulate the *ESR1* mediated renewal, proliferation, and differentiation of the pluripotent stem cells (Naugler et al., 2007[22]; Böser et al., 2013[5]; Sarachana and Hu., 2013[26]). On the other hand, *ESR1* and *ESR2* were accepted to take part in regulating autoimmunity in systemic lupus erythematosus by promoting the production of pathogenic autoantibodies (Bynoté et al., 2008[6]). Furthermore, the surrounding of *NCOA5* gene region where the *CD40* gene is also located includes possible susceptible genes of type 2 diabetes mellitus and rheumatoid arthritis (Raychaudhuri et al., 2008[25]; Lewis et al., 2010[20]). Recently, the significant relationship between the *NCOA5* rs2903908 polymorphism and susceptibility to psoriasis, which is a T cell mediated chronic inflammatory disease, was also reported (Zervou et al., 2011[32]). Additionally, genome-wide association studies on psoriasis and BD showed that these diseases share many common genetic risk factors (Lee et al., 2012[19]). Moreover, the *NCOA5* gene insufficiency leads to an elevated expression of Interleukin (IL)-6, and increased levels of IL-6 were found to be related to the pathogenesis of BD (Naugler et al., 2007[22]; Talaat et al., 2014[27]). These similarities led us to consider the *NCOA5* gene as a candidate for BD. Any association between the susceptibility and clinical outcomes of BD and the *NCOA5* rs2903908 polymorphism have not yet been clarified. Therefore, we aimed to clarify the impacts of the *NCOA5* rs2903908 polymorphism on susceptibility and clinical findings of BD. 

## Materials and Methods

### Sampling and genotyping

This study included 300 patients with BD and 371 ethnicity-matched healthy controls. All of the patients had been diagnosed according to the criteria of the International Study Group for BD (International Team for the Revision of the International Criteria for Behçet's Disease, 2014[16]). Informed consent was obtained from all participants before they enrolled in this study. The study was performed in accordance with the Declaration of Helsinki and was approved by the Ethics Committee of Gaziosmanpasa University, Faculty of Medicine. Patients, who had any autoimmune systemic inflammatory diseases, such as rheumatoid arthritis or genetic diseases, were excluded from the study. 

The age and gender data of the all participants are given in Table 1(Tab. 1). Clinical findings of oral aphthae, genital ulceration, skin lesions, uveitis, and vascular involvement of the patients were noted as present or absent. Skin lesions consisted of erythema nodosum or papulopustular lesions. 

We performed DNA purification with DNA isolation kits from peripheral venous blood samples (preserved in EDTA tubes) of the patient and control groups. Genetic analyses were performed by using the TaqMan allelic discrimination assay. All SNPs were genotyped in the same centre by using a TaqMan SNP genotyping assay in a StepOnePlus Real-Time Polymerase Chain Reaction (PCR) system by following the conditions recommended by the manufacturer (Applied Biosystems, Foster City, CA, USA). 

We examined the distribution of the *NCOA5* rs2903908 genotypes and alleles in all participants. For further evaluation, we investigated whether the *NCOA5* rs2903908 polymorphism had impacts on the development of any clinical findings such as genital ulceration, skin lesions, uveitis, or vascular involvement.

### Statistical analysis

Data were analyzed using the Statistical Package for Social Sciences (SPSS) software version 15.0 for Windows (SPSS Inc., Chicago, IL). Mean and standard deviation were used for the presentation of continuous quantitative variables. Frequencies and percentages were used for categorical data. The *NCOA5* rs2903908 overall genotype distribution was compared by chi-square (χ^2^) test, and the specific genotype and allele distributions were compared by using Fisher's exact test. The p-values smaller than 0.05 were considered significant. The odds ratios (ORs) and 95 % confidence intervals (CIs) were used to determine the relationships between the *NCOA5* allelic and genotypic variants and their occurrence in the patients. The *NCOA5* rs2903908 genotype distributions in both the patients and the healthy controls were analyzed according to the Hardy-Weinberg Equilibrium (HWE). 

## Results

In this study, a total of 300 unrelated Turkish patients with BD and 371 individuals without any established disease diagnoses were evaluated for the rs2903908 polymorphism of the *NCOA5* gene. The demographical and clinical findings of all participants are summarized in Table 1(Tab. 1). The mean age of the patients was 37.60 ± 8.85 years, and the mean age of the healthy controls was 37.00 ± 11.90 years. There was no significant difference in terms of age and gender between the BD patients and the healthy controls (p>0.05). Oral aphthae were seen in the majority of patients (98.48 %). 

The distributions of the genotypes and alleles of the patients and healthy controls for the *NCOA5* rs2903908 polymorphism are presented in Table 2(Tab. 2). The frequency of TT genotype of the *NCOA5* rs2903908 polymorphism was significantly higher in patients compared to those in healthy controls (p=0.016, OR=1.46, 95 % CI=1.08-1.99). We found significantly higher levels of the CT genotype of the *NCOA5* rs2903908 polymorphism in healthy controls compared to those in patients with BD (p=0.014, OR=0.66, 95 % CI=0.49-0.92) (Table 2(Tab. 2)). The genotype distribution of the rs2903908 polymorphism of the *NCOA5* gene in the patients and controls was compatible to HWE (p>0.05 for all) (data not shown). 

We also compared genotype and allele distribution of *NCOA5* rs2903908 polymorphism between patients and controls by gender basis, and we found statistical differences for CT and TT genotypes between female patients and controls (Table 3(Tab. 3)). In accordance with the general result, the CT genotype was higher in controls (p=0.014), and the TT genotype was higher in patients (p=0.026) (Table 3(Tab. 3)). 

The CT genotype of the *NCOA5* rs2903908 polymorphism was significantly higher in BD patients with genital ulceration (p=0.002, OR=3.03, 95 % CI=1.51-6.08), and the TT genotype was higher in BD patients without genital ulceration (p=0.014, OR=0.46, 95 % CI=0.25-0.84). Frequencies of C allele and genotypes including C allele (CC and CT) were significantly higher in the patients with uveitis compared to those without uveitis (p=0.0001, OR=2.19, 95 % CI=1.48-3.23 and p=0.0002, OR=2.49, 95 % CI=1.55-3.99, respectively) (Table 4(Tab. 4)). 

## Discussion

BD is an inflammatory disorder with recurrent oral aphthous ulcers, genital ulcers, and uveitis of which etiology and pathogenesis have not been fully elucidated (Gül, 2005[12]). Genetic analysis of the multicase families and populations demonstrated associations between BD and genetic factors. Whereas some authors reported human leukocyte antigen (HLA)-related genes as the predominant genetic risk factors for BD (Kirino et al., 2013[18]; Ortiz-Fernández et al., 2016[24]), others suggested several non-HLA genes (Chen et al., 2012[8]).

*NCOA5* is a nuclear protein with both coactivator and corepressor domains. It encodes a coregulator for ESR1, ESR2 and NR1D2 (Naugler et al., 2007[22]; Bento et al., 2008[4]; Lewis et al., 2010[20]). *NR1D2* was demonstrated to be upregulated in monocytes from psoriatic patients, and this upregulation enabled disease stage estimation with an accuracy rate of 86 % based on gene expression patterns (Hornung et al., 2002[13]). However, it was reported that *NR1D2* gene expression patterns from the peripheral blood of rheumatoid arthritis patients did not differ from that of healthy individuals (Naugler et al., 2007[22]). On the other hand, *NCOA5* deficiency resulted in elevated *IL-6* expression (Liu and Feng, 2014[21]). *NCOA5* is a known regulator of ESR1 and ESR2 which negatively regulate nuclear factor kappa B-induced IL-6 expression. *NCOA5* haploinsufficiency increases the IL-6 levels by disrupting ESR1 mediated repression of IL-6 transcription (Naugler et al., 2007[22]). Talaat et al. (2014[27]) showed that BD patients with active disease had significantly higher levels of IL-6 compared to patients in remission. In addition, many other genes indicated an association with BD (Chen et al., 2012[8]; Yazici et al., 2012[31]; Hou et al., 2013[14]; Kirino et al., 2013[18]; Tasliyurt et al., 2013[28]; Tizaoui et al., 2014[29]; Xiang et al., 2014[30]). However, none of these genes are fully linked to BD.

Because *NCOA5* can affect many factors and pathways, and it can work both as a coactivator and corepressor, perhaps *NCOA5* may have an impact on BD, especially in females, by a way that is not yet undisclosed. This hypothesis was supported by Gao et al. who showed that the deletion of both IL-6 alleles does not completely block hepatocellular carcinoma (HCC) development in *NCOA5*+/ male mice, and other downstream targets of *NCOA5* may also contribute to hepatocarcinogenesis (Gao et al., 2013[10]).

Previously, heterozygous deletion in the *NCOA5* gene was found to be related to increased susceptibility to both glucose intolerance and HCC, partially by elevated IL-6 expression (Gao et al., 2013[10]). Conversely, *NCOA5* polymorphisms, including rs2903908, were reported not to be related to type 2 diabetes mellitus (Lewis et al., 2010[20]). However, in a genome-wide association study of type 2 diabetes mellitus, the results of *NCOA5* gene polymorphisms were conflicting (Bento et al., 2008[4]). Moreover, in the present study, significant relationship between the *NCOA5* rs2903908 polymorphism and BD were found. Namely, we found that TT genotype and the T allele of the *NCOA5* rs2903908 polymorphism were related to an increased (approximately one and half-fold) susceptibility to BD and it has a protective role for genital ulceration and uveitis in this study. Also, the CT genotype of the *NCOA5* rs2903908 polymorphism was found to be associated with an increased development of uveitis (approximately two-fold) and genital ulceration (approximately three-fold) as well as to have a protective role on the development of BD (Table 4(Tab. 4)). Due to overexpression of IL-6 associated with genital ulceration and uveitis (Talaat et al., 2014[27]), and considering the results of recent studies that *NCOA5* haploinsufficiency is causing an increase in IL-6 expression (Sarachana and Hu, 2013[26]; Gao et al., 2013[10]), the CT genotype for rs2903908 polymorphism may also be causing *NCOA5* haploinsufficiency. The rs2903908 polymorphism is a T to C transition in the intronic region of the* NCOA5* gene. In the literature, we could not find any studies showing the effect of the rs2903908 polymorphism on the expression of *NCOA5* gene. A variant in intronic region may form a cryptic splice site that enhance the use of that site by making it more similar or identical to the normal splice site. Therefore, we can consider that the C allele reduces the expression of *NCOA5*. However, confirmation of this hypothesis is needed, especially when considering why the CC genotype is not significantly higher in patients with genital ulcers and uveitis. Inconsistent with our results, Zervou and colleagues (2011[32]) reported significantly higher levels of the CC genotype and the C allele of the *NCOA5* rs2903908 polymorphism in patients with psoriasis, and they emphasized that the CC genotype and the C allele of the *NCOA5* rs2903908 polymorphism might be related to an elevated susceptibility to psoriasis (Zervou et al., 2011[32]). Our study did not find a significant difference for the CC genotype between the patients and the control group. These discordant results may be due to the various underlying pathogenetic mechanisms of the diseases, as well as different ethnic basis and environmental factors. The study of Gao et al. (2013[10]) showed that the *NCOA5* gene may be impacted positively and negatively by more factors than we previously mentioned. Nevertheless, this discrepancy may be clarified by future studies investigating the impacts of the *NCOA5* rs2903908 polymorphism on several chronic inflammatory diseases. 

To the best of our knowledge, this preliminary study represents, for the first time, the association between the *NCOA5* rs2903908 polymorphism and the susceptibility and clinical findings of BD. We arrived at three main results. First, the frequency of TT genotype of the *NCOA5* rs2903908 polymorphism was significantly higher in the female BD patients compared to those in the healthy controls, and this genotype and allele seemed to be related to an increased (approximately one and half-fold) risk of BD development (p=0.026, OR=1.67, 95 % CI=1.08-2.59). In addition, the CT genotype of the *NCOA5* rs2903908 polymorphism appeared to prevent individuals from developing BD. Second, the CT genotype of the *NCOA5* rs2903908 polymorphism seemed to predispose the BD patients to develop both genital ulceration and uveitis more frequently (approximately three-fold for genital ulceration and two-fold for uveitis), whereas the TT genotype of *NCOA5* rs2903908 polymorphism was found to have protective impacts on developing genital ulceration or uveitis in the patients with BD. Finally, we found that the BD patients who are carrying the C allele at the *NCOA5* rs2903908 locus may be more prone to developing uveitis. Although CC genotype was observed 3 times more in patients with uveitis than without uveitis, the statistically significance was not found (8.24 % vs. 2.91 %; p=0.070, OR=2.80, 95 % CI=0.93-8.23) (Table 4(Tab. 4)).

In conclusion, our study identified that the *NCOA5* rs2903908 polymorphism seemed to be linked to BD susceptibility in females and in clinical findings. Additionally, the *CD40 *gene, which is located near the region of the *NCOA5* gene, was previously related to several chronic autoimmune inflammatory diseases. We conclude that the *NCOA5* gene may be a good candidate for future studies investigating genetic tendencies for chronic inflammatory and autoimmunity diseases that share similar pathogenic pathways. How the rs2903908 variant affects the *NCOA5* mRNA or protein expression and function and how this polymorphism influences the pathogenesis of BD and its symptoms remain mysteries to be solved. 

## Acknowledgements

The manuscript was professionally edited by www.scribendi.com. We thank Osman Demir for his help in statistical analyses. 

The study was supported by a grant from Scientific Research Projects of Gaziosmanpasa University, project number #2012/20.

## Declaration of interest

The authors report no declarations of interest. 

## Figures and Tables

**Table 1:**
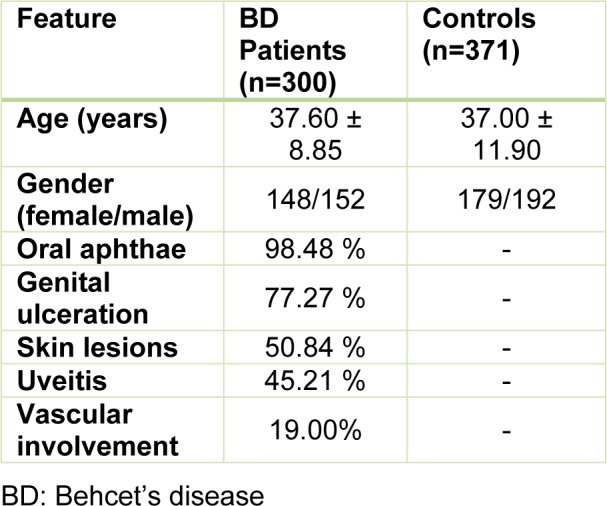
The demographical and clinical findings of all participants

**Table 2:**
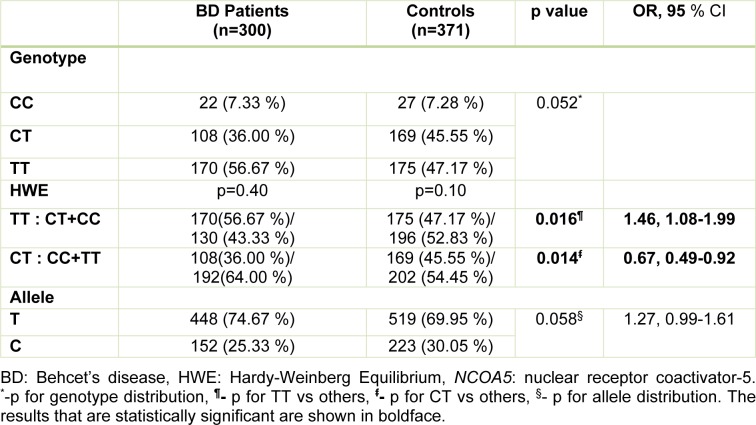
The distribution of genotypes and alleles of *NCOA5* rs2903908 polymorphism in patients and controls

**Table 3:**
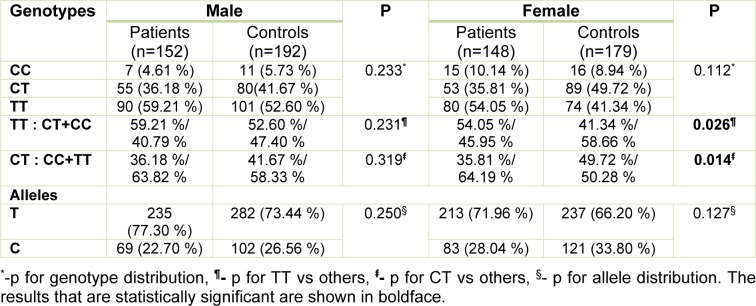
The distribution of genotypes and alleles of *NCOA5* rs2903908 polymorphism in patients and controls by the gender basis

**Table 4:**
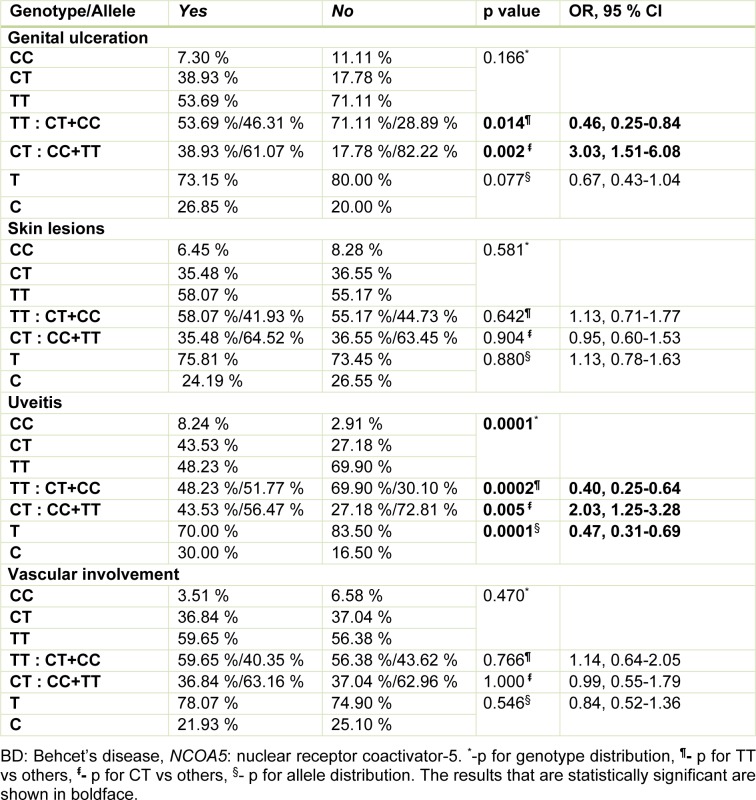
The distribution of genotypes and alleles of *NCOA5* rs2903908 polymorphism in BD patients with and without genital ulceration, skin lesions, uveitis and vascular involvement
